# Preparation and Characterization of Nanofiber Coatings on Bone Implants for Localized Antimicrobial Activity Based on Sustained Ion Release and Shape-Preserving Design

**DOI:** 10.3390/ma17112584

**Published:** 2024-05-28

**Authors:** Yubao Cao, Hong Wang, Shuyun Cao, Zaihao Liu, Yanni Zhang

**Affiliations:** 1 School of Machinery and Automation, Weifang University, Weifang 261061, China; 2 College of Mechanical and Electronic Engineering, Shandong University of Science and Technology, Qingdao 266590, China; 3 Key Laboratory for Space Bioscience and Biotechnology, School of Life Sciences, Northwestern Polytechnical University, Xi’an 710072, China

**Keywords:** Cu-doped SNW, shape-preserving, bond strengths, sustained ion release, antibacterial activity

## Abstract

Titanium (Ti), as a hard tissue implant, is facing a big challenge for rapid and stable osseointegration owing to its intrinsic bio-inertness. Meanwile, surface-related infection is also a serious threat. In this study, large-scale quasi-vertically aligned sodium titanate nanowire (SNW) arrayed coatings incorporated with bioactive Cu^2+^ ions were fabricated through a compound process involving acid etching, hydrothermal treatment (HT), and ion exchange (IE). A novel coating based on sustained ion release and a shape-preserving design is successfully obtained. Cu^2+^ substituted Na^+^ in sodium titanate lattice to generate Cu-doped SNW (CNW), which maintains the micro-structure and phase components of the original SNW, and can be efficiently released from the structure by immersing them in physiological saline (PS) solutions, ensuring superior long-term structural stability. The synergistic effects of the acid etching, bidirectional cogrowth, and solution-strengthening mechanisms endow the coating with higher bonding strengths. In vitro antibacterial tests demonstrated that the CNW coatings exhibited effective good antibacterial properties against both Gram-positive and Gram-negative bacteria based on the continuous slow release of copper ions. This is an exciting attempt to achieve topographic, hydrophilic, and antibacterial activation of metal implants, demonstrating a paradigm for the activation of coatings without dissolution and providing new insights into insoluble ceramic-coated implants with high bonding strengths.

## 1. Introduction

Titanium and its alloys are used in the repair and replacement of human hard tissue and have become the preferred metal materials for replacement or repair products such as artificial joints, artificial bones, and broken bone fixators. However, as bioinert material titanium can easily encapsulate fibrous tissue in the early stages of implantation, this makes it difficult to form a firm osseous bond with the host bone [1]. Currently, the application of implant materials is mainly limited by the lack of proper interaction between the materials and bone tissue, which is characterized by the difficulty of bone integration and the serious shortening of the service life of implants [2].

Studies have shown that the interface between the tissue and the implant material is a key factor in determining the effect of bone integration. As the most important aspect of bone implants, this interface can enhance the functional activities of osteoblasts, inducing solid contact between the implants and bone tissue without fibrous connective tissue [3]. The main parameters that determine the interface properties of tissue and implant materials include the chemical composition of the implant surface [4,5,6], surface energy or hydrophilicity [4,7], roughness [8,9], surface morphology [4,10,11,12,13], and mechanical properties of the coating [14]. Nanofiber scaffolds with three-dimensional structures have attracted increasing attention because they more closely resemble the natural cell-growth microenvironment [15,16]. The surface topology of nanofiber coatings can promote the exploration, sensing, and migration of cells, enabling more actin cytoskeleton fibers to be synthesized into platelike pseudopods, and form a firm bond with the material surface [17,18,19]. The surface of a nanofiber network absorbs four times more serum proteins than the smooth surface and enhances the adhesion of cells [20], which reflects the high biological activity of the nanofiber network. Therefore, bioactivation modification on titanium metal is the key to improving the repair and replacement of hard tissue.

Although biomimetic coatings can be obtained by gel–sol, magnetron sputtering, micro-arc oxidation, vapor deposition, and plasma spraying, these coatings are neither nanofiber forms nor can they be firmly bonded to the matrix [21,22]. Wear and spalling occur in the process of implantation owing to poor binding, and aseptic loosening is further caused by tiny particles produced by spalling.

Currently, many implants constructed with surface topological configurations dissolve and collapse during physical/chemical reactions with the surrounding microenvironment after implantation, which does not guarantee the subsequent regulation of cell behavior [23,24]. To resolve the conflict between early osseointegration and long-term fixation of soluble ceramic-coated implants to bone, persistent efforts to seek alternative strategies to dissolution-derived activation are currently being conducted. In particular, nanostructured titania [25], titanate nanotubes [26], and nanofibers [27], all of which are insoluble, have been recently developed to act as hydrophilic and topographic activators of Ti implants, creating a paradigm for activation without the dissolution of coatings.

It is increasingly recognized that an ideal bone integration process also requires good neovascularization, which not only provides nutrition supply for new bone formation, but also promotes the remote homing of bone-marrow-derived stem cells (MSCs) to the implant surface to form bone [28]. Copper is an essential trace element in the human body that participates in various metabolic processes [29]. Meanwhile, copper ions can promote angiogenesis and osteogenesis [30,31,32], accelerate the early healing of skin wounds [33,34,35], and exhibit good antibacterial properties by inactivating bacterial central catabolism and biosynthesis modes [31,32,35,36]. Compared with the antibacterial metal ions such as zinc and silver ions, copper ions show the best compromise between antibacterial action and cytotoxicity [32].

Herein, considering morphology bionics and inorganic active metal ion loading, we design and construct copper-doped titanate nanowires coatings (CNWs) with high binding strength and stability. Moreover, benefiting from the slow-release of Cu^2+^ ions, the obtained coatings exhibit superior antibacterial activity, against both Gram-positive and Gram-negative bacteria, indicating bright prospects in biomedical implant applications.

## 2. Materials and Methods

### 2.1. Preparation and Cu^2+^ Ionic Incorporation in SNW Coatings

First of all, the commercial pure titanium rod (purity of 99.5 wt%) is processed into titanium sheets (φ14 × 2 mm) and then polished with 320–1500 metallographic sandpaper in turn, and finally mechanical polishing with diamond grinding paste to achieve the mirror bright effect [26]. The pitting surface was obtained by etching the mechanical polished Ti disks (P-Ti) for 25 s at room temperature to remove surface oxides and dirt. The etching solution consisted of 1:1:8 nitric acid (HNO_3_, 69.2%; Sinopharm Chemical Reagent Co., Ltd., Shanghai, China), hydrofluoric acid (HF, 40%; Sinopharm Chemical Reagent Co., Ltd., Shanghai, China), and deionized water. The acid-etched Ti disks (A-Ti) were ultrasonically cleaned in acetone, ethanol, and distilled water and dried in an ambient atmosphere. For the topographical SNW growth, each A-Ti disk was hydrothermally treated in 20 mL 1 M aqueous NaOH (Sinopharm Chemical Reagent Co., Ltd., Shanghai, China) at 220 °C for 2.5 h. After the reaction vessel cooled to room temperature, the obtained SNW-coated A-Ti (referred to as SNW/A-Ti) disks were removed and hydrothermally treated in 20 mL 1 mM aqueous CuCl_2_·2H_2_O (Sinopharm Chemical Reagent Co., Ltd., Shanghai, China) at 150 °C for 2 h for Cu^2+^ ion incorporation. The resulting samples are referred to as CNW/A-Ti.

### 2.2. Structural Characterization of the Coatings

The morphologies of the samples including surface and cross-section were observed using field-emission scanning electron microscopy (MERLIN COMPACT, Carl Zeiss AG, Cambridge, UK). From these, ImageJ software (v 1.37c, National Institutes of Health, Bethesda, MD, USA) was used to examine the nanowire diameter and length. Five random areas were measured to obtain average values. The elemental compositions of the samples were examined by energy-dispersive X-ray spectrometry (X-MamN, Oxford, UK). X-ray diffractometry (X’Pert PRO, PANalytical Co., Almelo, The Netherland) was used to identify phasic components. (Cu-Kα, 20–55°, 40 kV, a scanning speed of 3° min^−1^ and an incident angle of 1°). X-ray photoelectron spectroscopy (K-Alpha, Thermo Scientific, Waltham, MA, USA) was used to detect the chemical species on the surfaces of the coatings (Al-Kα and a photoelectron takeoff angle of 45°). The binding energy of 284.8 eV (C 1s) was used to calibrate the XPS spectra obtained. The atomic force microscopy (SPM-9500J3, Shimadzu, Kyoto, Japan) was used to perform surface roughness measurements. Five random areas were measured to obtain average values.

### 2.3. Bonding Strengths of the Coatings

To identify the adhesive strengths of the coatings on the Ti discs, scratch tests were performed using an auto-scratch coating tester (WS-2006, Zhongke Kaihua Technology Development Co., Ltd., Lanzhou, China) that consisted of a spherical stylus [37]. According to the set loading rate and scratch speed, the computer control system starts continuous loading and translation of the measured sample at the same time. With the increasing load applied from the tip of the diamond cone to the surface of the sample and the continuous movement of the sample, the changing load value during the scratch process and the acoustic emission signal generated when the coating on the surface of the sample is damaged and spalling are automatically detected. The corresponding curve of acoustic emission signal and load change is shown. When the set load is reached, the test ends. The loading load is automatically unloaded to zero and the sample is returned to its original position. The load corresponding to the initial identifiable fault location is called the critical load (Lc). It is determined by acoustic emission signal and SEM microregion analysis of the scratches. Three samples were used for each coating type, and the critical loads were averaged.

### 2.4. Wettability of the Coatings

The hydrophilicity of the coatings can be expressed by the water contact angle, which was measured using a surface contact angle measurement machine (DSA30, Kruss, Berlin, Germany) [38]. Five samples were used for each coating type, and the measurements were averaged.

### 2.5. Cu^2+^ Released from the Coatings

The in vitro release of Cu^2+^ ions from the coatings was evaluated by immersing them in physiological saline (PS) solutions (e.g., 0.9 wt% aqueous NaCl) at 37 °C for 0, 1, 3, 7, 14, and 21 d, and then examining the changes in ion concentrations of the obtained solutions by inductively coupled plasma emission spectroscopy (ICAP 6300, Thermo Scientific, Waltham, MA, USA) [39]. At the same time, scanning electron microscopy was used to observe the surface and cross-section morphology of the immersion coating. Five specimens of each coating were used at each immersion time.

### 2.6. In Vitro Antibacterial Test

In the antibacterial test in vitro, *Staphylococcus aureus* (*S. aureus* ATCC25293, China General Microbiological Culture Collection Center) was used as Gram-positive bacteria model and *Escherichia coli* (*E. coli* ATCC25922, China General Microbiological Culture Collection Center) as Gram-negative bacteria model. The sample was placed in a 24-well plate, each well was injected with 1 mL bacterial solution at a concentration of 1 × 10^6^ CFU·mL^−1^. The samples were washed at least 3 times in PBS after incubation at 37 °C for 24 h. The bacteria attached to the samples were separated in 1 mL PBS by ultrasonic vibration for 5 min [40]. The isolated bacteria were collected and inoculated into an agar culture medium. The viable bacteria were counted by the Chinese National Standard (GB/T 4789.2 [41]) after incubation at 37 °C for 24 h. The calculation formula of antibacterial rate is expressed as Ra (%) = (B − A)/B × 100%, where A and B are the number of viable bacteria on the coatings and A-Ti, respectively. Each test was repeated four times.

## 3. Results

### 3.1. Topography of the P-Ti before and after Acid Etching

**Figure 1:**
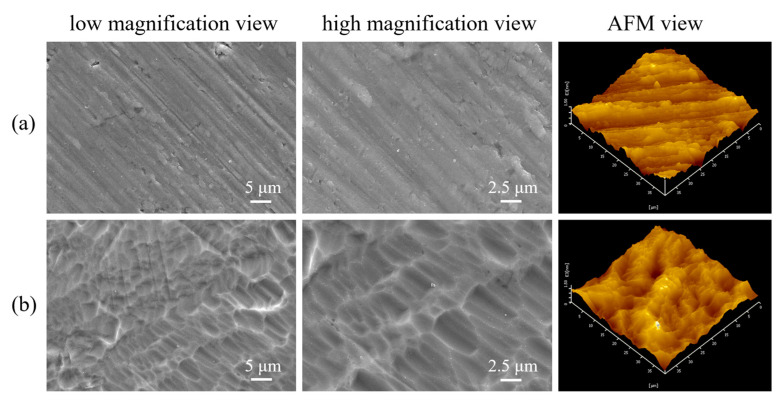
Surface SEM and AFM images of (**a**) P-Ti and (**b**) A-Ti.

The surface SEM and AFM images are shown in Figure 1 for P-Ti disks before and after acid etching. A homogeneous micro-pitted structure was produced on the titanium matrix of A-Ti by acid-etching that can also make the titanium matrix fully exposed, clean, and uniform. AFM images of the specimens are shown on the right-hand side of Figure 1. A rougher surface has a larger surface area, which is more conducive to cell adhesion, proliferation, and differentiation in vitro and early bone growth in vivo [32,42,43,44,45]. Based on a 40 × 40 μm^2^ scan area, P-Ti had a plain structure with Ra (average surface centerline roughness), Rq (root mean square roughness), and Rz (10-point height of irregularity roughness) values of 141.1 ± 7.4, 181.1 ± 4.7, and 1074.0 ± 97.0 nm, respectively. A-Ti exhibited a microdimple-patterned Ti surface with Ra, Rq, and Rz values of 205.9 ± 12.8, 256.3 ± 13.8, and 1180.9 ± 113.4 nm, respectively. This reveals that the surface characteristics of A-Ti are superior to those of P-Ti. The clean and simple reaction interface is not only conducive to the subsequent hydrothermal reaction but also to the high adhesion of the coatings on the Ti substrates. At the same time, the micro-pitted structure enables the coatings to be embedded with the substrate through the “rivet” effect, that is, to further increase the binding between the substrate and the coatings through mechanical embedment.

### 3.2. Structure and Topography of the HT Coatings before and after IE

**Figure 2:**
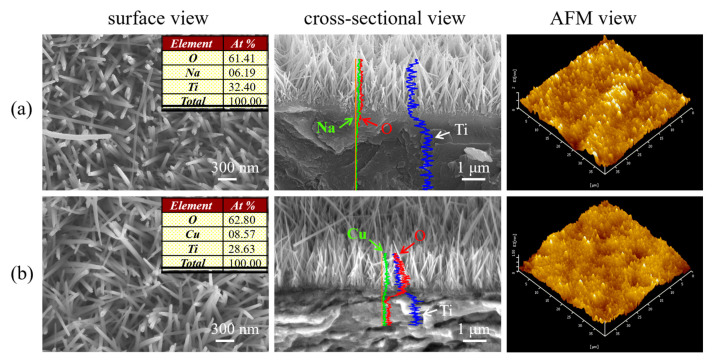
Surface, cross-sectional SEM morphologies, and AFM images of the as-synthesized (**a**) SNW/A-Ti and (**b**) CNW/A-Ti.

Figure 2 shows the SEM and AFM images of SNW/A-Ti and CNW/A-Ti. The surface morphologies of all coatings exhibited well-ordered structures. The A-Ti discs after the reaction with alkaline under hydrothermal conditions are shown on the left of Figure 2a. Large-scale quasi-vertically aligned and top-flat SNW (diameter 54.2 ± 2.8 nm, length approximately 2.2 µm) formed uniformly on the A-Ti substrates. After hydrothermal IE with 0.001 M CuCl_2_ at 150 °C for 2 h, CNW with a diameter of 54.8 ± 1.7 nm and a length of approximately 2.1 µm formed, as shown on the left of Figure 2b. It can be observed that copper incorporation does not alter the main structural characteristics, including the nanowire morphology and size. According to the EDX results presented in the tables (inserted on the left images in Figure 2), Na, Ti, and O are present on the SNW/A-Ti surface, whereas, after IE, only Cu, Ti, and O are detected on the CNW/A-Ti surface, demonstrating the complete replacement of Na^+^ ions by Cu^2+^ ions. We achieved uniform incorporation of Cu^2+^ ions into the SNW throughout the entire length by analyzing cross-sectional SEM morphologies and EDX profiles as shown in Figure 2 (the center images). There was no apparent discontinuity between the coatings and the Ti substrates for either SNW/A-Ti or CNW/A-Ti, indicating that all the coatings were tightly bonded to the Ti substrates. From the AFM images (right side of Figure 2), SNW/A-Ti exhibited a dual-scale topography that appeared to be micro-concave with overlapping quasi-vertically aligned SNWs. The Ra, Rq, and Rz values of SNW/A-Ti were slightly greater than those of A-Ti. Moreover, the measured roughness values (Table 1) had no significant difference in between SNW/A-Ti and CNW/A-Ti. The similar nanowire topographies suggest that the incorporation of Cu into the sodium titanate did not significantly alter the surface topographical characteristics. Thus, the Na^+^ ions in the SNW could be thoroughly eliminated via a facile ion-exchange strategy with the desired Cu^2+^ ions, and the surface nanowire topography on the titanium substrate was simultaneously retained.

### 3.3. Phases and Surface Chemical Species of the Coatings

**Figure 3:**
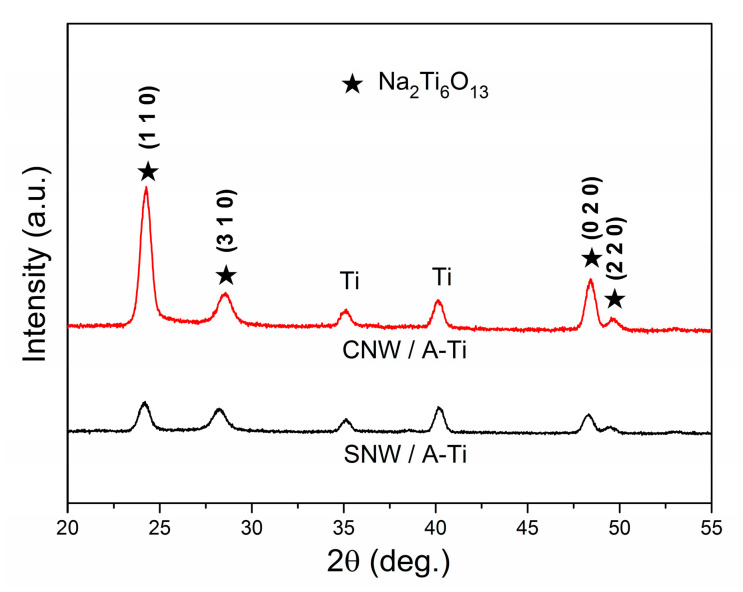
XRD patterns of the coatings.

The phase components of the samples were analyzed using an X-ray diffractometer, the XRD patterns detected are shown in Figure 3. The peaks (24.2°, 28.22°, 48.32°, and 49.52°) correspond to the crystallographic planes of Na_2_Ti_6_O_13_ phase (JCPDS no. 73-1398), which are (1 1 0), (3 1 0), (0 2 0), and (2 2 0), respectively. For CNW/A-Ti, none of the Cu-containing phases were detected. Compared with SNW/A-Ti, no obvious changes were observed in the phase components, only the peak positions were shifted slightly to higher angles. In the monoclinic Na_2_Ti_6_O_13_ lattice, the substitution of Cu^2+^ (0.72 Å) causes lattice contraction due to smaller ionic radius than Na^+^ (0.95 Å).

**Figure 4:**
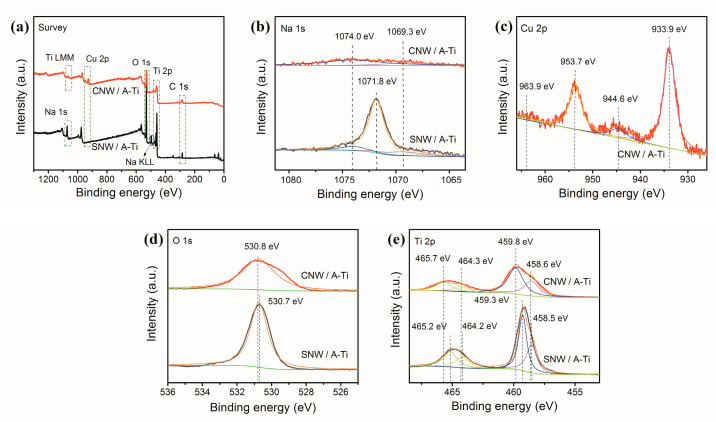
XPS spectra of the coatings: (**a**) full spectra, (**b**) Na 1s, (**c**) Cu 2p, (**d**) O 1s, and (**e**) Ti 2p.

The surfaces of the coatings were analyzed using XPS to explore the chemical states of the elements. Figure 4a shows the full spectra of SNW/A-Ti and CNW/A-Ti, revealing that the high Na content in SNW/A-Ti decreased to nearly zero upon conversion to CNW/A-Ti via copper IE, with an associated increase in the Cu content. This result can also be drawn from the appearance of the peak at ~506.4 eV for SNW/A-Ti, which could be related to Na KLL Auger electrons. However, it is not detected in CNW/A-Ti.

The high-resolution XPS spectra of Na 1s, Cu 2p, O 1s, and Ti 2p are shown in Figure 4b–e, respectively. The Na 1s spectra of SNW/A-Ti and CNW/A-Ti (Figure 4b) show that the peaks at 1074.0 and 1069.3 eV could be related to two Ti LMM Auger electron peaks, respectively [46]. The peak at 1071.8 eV was assigned to Na^+^ (Na 1s) in SNW/A-Ti [47,48]. This peak was not detected for CNW/A-Ti.

In the Cu 2p spectra (Figure 4c), the peaks at 933.9 eV (Cu 2p_3/2_) and 953.7 eV (Cu 2p_1/2_), and the existence of the doublet shakeup satellites at 944.6 and 963.9 eV, respectively, indicate that Cu bonds with O and exists in divalent ion states in the CNW coatings [49]. The disappearance of the peak at 1071.8 eV (Na^+^ in the Na 1s spectra) and ~506.4 eV (Na KLL Auger electrons in the full spectra) and the existence of the characteristic peaks of Cu^2+^ indicate that Na^+^ ions were nearly completely replaced by Cu^2+^ during the IE reaction.

In the O 1s spectrum (Figure 4d), the peak at 530.7 eV for SNW/A-Ti, and 530.8 eV for CNW/A-Ti are corresponding to O atoms bound to metal atoms, respectively [50].

The Ti 2p spectra (Figure 4e) show four peaks for each sample. The peaks at 458.5 and 459.3 eV for SNW/A-Ti, and 458.6 and 459.8 eV for CNW/A-Ti could be attributed to Ti 2p_3/2_ Ti(Ⅲ) and Ti(Ⅳ), respectively. The peaks at 464.2 and 465.2 eV for SNW/A-Ti and 464.3 and 465.7 eV for CNW/A-Ti could be ascribed to Ti 2p_1/2_ Ti(Ⅲ) and Ti(Ⅳ), respectively [46,50,51]. Compared with the Cu-free sample, the CNW/A-Ti had a shift toward higher binding energies about 0.1–0.5 eV.

Na_2_Ti_6_O_13_ is composed of four TiO_6_ octahedral units that share edges on a single level. These units are combined with several similar units above and below through additional edge-sharing [52]. The presence of Cu^2+^ ions can replace the Na^+^ ions in the titanate layer via ion exchange (IE). After Cu doping, the Ti 2p peaks shift toward higher binding energies due to the electronic interaction between incorporated Cu^2+^ and TiO_6_ layers. Based on the combined results from EDS, XRD, and XPS analyses, successful Cu doping of CNW/A-Ti can be confirmed along with the existence of Cu in the form of Cu^2+^.

### 3.4. The Formation Mechanism of SNW/A-Ti and CNW/A-Ti

The formation mechanism of novel nanostructures on titanium metal can be explained by considering the simultaneous processes of dissolution, reprecipitation, and subsequent IE. According to the experimental results presented herein and the literature reported, the chemical reaction for the synthesis of the titanate ions (${HTiO}_{3}^{-}$) can be described by the following formula (Equations (1)–(3)). When pure titanium is placed in an alkali solution at high temperature and pressure, the hydroxyl group in solution will react with it (Reaction 1) [53,54]:

(1) $$Ti+3{OH}^{-}\to {Ti(OH)}_{3}^{+}+4e^{-}$$

Therewith the hydrated titanium dioxide is obtained by ${Ti(OH)}_{3}^{+}$ (Reaction 2):

(2) $$Ti({OH)}_{3}^{+}+e^{-}\to {TiO}_{2}H_{2}O+0.5H_{2}$$

According to Reaction 3, the hydroxyl group will further attack the hydrated titanium dioxide to generate titanate ions (${HTiO}_{3}^{-}$) with negatively charged surfaces developing affinities for metal cations [55]:

(3) $${TiO}_{2}H_{2}O+{OH}^{-}\to {HTiO}_{3}^{-}+H_{2}O$$

The ${HTiO}_{3}^{-}$ anions formed in the dissolution step react with Na^+^ cations in an alkaline solution to form Na_2_Ti_6_O_13_ [56,57,58,59]. This precipitation reaction can be represented as:

(4) $$2{Na}^{+}+6HTiO_{3}^{-}\to {Na}_{2}{Ti}_{6}O_{13}+H_{2}O+4OH^{-}$$

After immersion in an aqueous CuCl_2_ solution, the Cu^2+^ ions with a smaller radius than Na^+^ ions could more easily diffuse into the inter-lattice space (7.466 Å) of the Na_2_Ti_6_O_13_ coating, replacing the latter.

Compared to the SNW, the incorporation of Cu^2+^ ions increases lattice distortion and decreases interplanar distances. However, the surface morphology and phase of the CNW remain unchanged.

### 3.5. Hydrophilicity of Coatings

**Figure 5:**
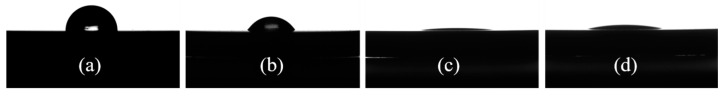
Water contact angle images of (**a**) P-Ti, (**b**) A-Ti, (**c**) SNW/A-Ti, and (**d**) CNW/A-Ti.

The hydrophilicity of hard-tissue implants is crucial for osteointegration in the biological tissue response usually indicated by the water contact angle. Figure 5 shows the images of water droplets on P-Ti, A-Ti, SNW/A-Ti, and CNW/A-Ti; the corresponding contact angles are 95.7 ± 1.15°, 57.3 ± 0.16°, 8.2 ± 1.12°, and 14.3 ± 0.76°, respectively. Acid etching and further arraying with SNWs improved the surface energies and, thus, the hydrophilicities of A-Ti and SNW/A-Ti compared to those of P-Ti. Although there was no significant difference in the surface topographical characteristics of SNW/A-Ti and CNW/A-Ti, according to the structural analysis described above, the contact angles of CNW/A-Ti were slightly greater than those of SNW/A-Ti.

### 3.6. Bonding Integrity of the Coatings

**Figure 6:**
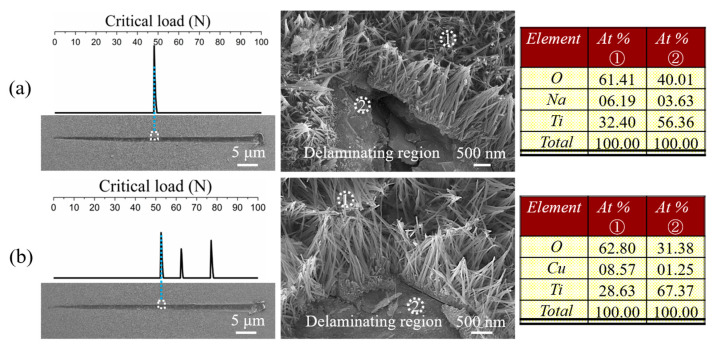
Curves of acoustic output versus load and scratch morphologies of (**a**) SNW/A-Ti and (**b**) CNW/A-Ti, including magnified views of initially delaminating areas along with EDX values detected on the ①—marked nanowires and ②—marked delaminating region.

We used a scratch test to characterize the bond strength of a coating by the critical load (Lc), depending on the failure occurring at the coating/substrate interface or in the interior of the coating under this load. The acoustic output vs. load curves and scratch morphologies of the coatings are shown in Figure 6. The initially delaminating areas occurred in the interiors of the coatings, indicating that the critical loads characterized the cohesive strengths of the coatings and that the adhesive strengths between the coatings and substrates were higher than the Lc values. This is supported by the fact that the SNW-traced Na and the CNW-traced Cu could still be detected by EDX in the delaminating region, where the Na and Cu contents were far lower than those detected on the unscratched coating surfaces (listed on the right of Figure 6). As shown on the left of Figure 6, the cohesive strengths of SNW/A-Ti and CNW/A-Ti are 48.5 ± 0.48 and 52.6 ± 0.53 N, respectively, indicating a strong binding of the coatings to the substrates.

This exciting bond strength of titanate coatings has not been previously achieved [60,61,62,63] and may be due to the contribution of three mechanisms: acid etching, bidirectional cogrowth, and solution strengthening. As discussed in Section 3.1, before the HT, an acid etching process was implemented, which further removed dirt and oxides from the surface of P-Ti and fully exposed the fresh titanium matrix to react with the hydrothermal solution. Because the interface is clean as well as micro-pitted, the hydrothermal SNW coatings are embedded with the substrate through the “rivet” effect, that is, the adhesive strength between the coating and substrate is further increased through mechanical embedment. Dong et al. [27] studied the downward/upward bidirectional cogrowth of approximately vertical titanate nanowire coatings on a Ti substrate in a NaOH solution, during which a corrosion region emerged on the Ti surface and grew downward, forming a dense base for titanate nanowires to root deep inside. Therefore, accompanied by the in situ downward growth of titanate nanowires, the cogrowth mechanism may lead to improved adhesive strength between the titanate coatings and the Ti substrate. Compared with the SNW, the critical load of the CNW is further increased, which can be attributed to the lattice distortion of the SNW caused by the replacement of Na^+^ by Cu^2+^ during IE, which increases the deformation resistance, leading to solid solution strengthening and thus increases the cohesion strength of the CNW. Therefore, the synergistic effect of the acid etching, bidirectional cogrowth, and solution-strengthening mechanisms can endow the coating with higher adhesive and cohesive strengths.

### 3.7. Cu^2+^ Release of the Coatings

**Figure 7:**
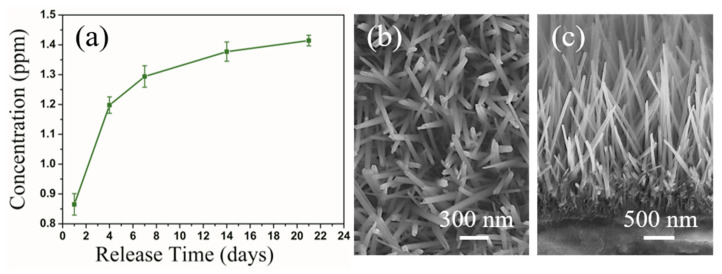
(**a**) Cu^2+^ release kinetics from CNW coatings in PS solution for different immersion times and their (**b**) surface and (**c**) cross-sectional SEM images after immersion for 21 d.

Figure 7 shows the quantification of the Cu^2+^ ions released from the CNW coatings immersed in PS solutions for 1, 3, 7, 14, and 21 d. The Cu^2+^ concentrations in the PS solutions after immersing the coatings tended to increase sharply in the period of 0–7 d and then gradually stabilized (Figure 7a). All samples exhibited a sustained release of ions during the three weeks. Moreover, the surface and cross-sectional morphologies (Figure 7b,c) of the coatings immersed in PS for 21 d showed no changes compared to the non-immersed ones. These results suggest that the coatings have good structural stability. The Cu^2+^ ions substitute Na^+^ ions in the SNW and can be efficiently released from the lattice via exchange with Na^+^ ions from the solution rather than dissolution, endowing the coatings with superior long-term structural stability and bond strength. Over the prolonged immersion process, Cu^2+^ ions were initially rapidly released and then continuously at a low level. The cumulative release from 1 to 21 days is between 13.6 ± 0.5 and 22.3 ± 0.3 μM. According to the literature, Cu^2+^ ions do not damage mammalian cells within the concentration range of 10–100 μM [63,64,65], and they can simultaneously promote angiogenesis [30,31] and osteogenesis [32].

### 3.8. Bactericidal Effects of the Coatings

**Figure 8:**
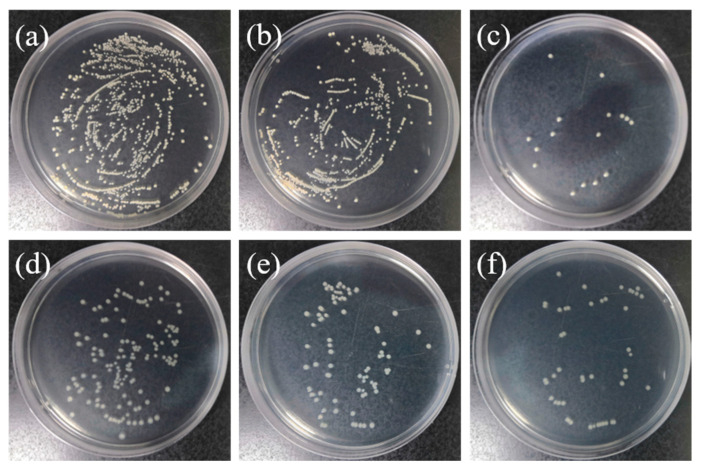
Bacterial colonies on agar culture plates of (**a**–**c**) *S. aureus* and (**d**–**f**) *E. coli* cultured on various samples: (**a**,**d**) A-Ti, (**b**,**e**) SNW/A-Ti, and (**c**,**f**) CNW/A-Ti.

The bactericidal effects of the coatings against adhesive *S. aureus* and *E. coli* were assessed by counting the bacterial colonies formed by dissociating them from the coated discs and re-cultivating them on agar, as shown in Figure 8. Compared to A-Ti, SNW/A-Ti and CNW/A-Ti exhibited antibacterial rates of 42.28 ± 3.7% and 69.13 ± 5.1% toward *E. coli*, respectively, and 42.71 ± 1.9% and 97.22 ± 1.4% toward *S. aureus*. Significantly fewer live bacteria were observed to adhere to SNW/A-Ti, indicating surface nanowire topography without additional contribution to bacterial adhesion, despite its higher surface roughness values compared to those of A-Ti. Furthermore, the number of viable bacteria decreased further for CNW/A-Ti, indicating pronounced bactericidal activity derived from the incorporation of Cu^2+^ ions. Cu^2+^ can damage respiratory enzymes by generating reactive oxygen species, and cause cell lysis, cytoplasm leakage, and bacterial death by extracting electrons from bacterial membranes [31,66]. The CNW coatings exhibited good antibacterial properties against Gram-positive and Gram-negative bacteria for localized antimicrobial delivery based on the continuous slow release of copper ions. These results suggest that coatings incorporated with bioactive Cu^2+^ ions may give rise to an advanced implant of improved clinical performance.

## 4. Discussion

The CNW coatings were grown hydrothermally on the surface of Ti disks where Cu^2+^ ions substituted Na^+^ in sodium titanate lattice and were efficiently released from the structure in a PS solution. Copper incorporation did not alter the micro-structure or phase components of the SNW coatings, ensuring superior long-term structural stability and bond strength. With the presence of released Cu^2+^ ions, the CNW-coated Ti disks (CNW/A-Ti) exhibited significant contact killing activity against *S. aureus* and *E. coli*. Therefore, this successful development presents a novel coating design that combines good adhesion strengths to Ti substrates with localized antimicrobial delivery and shape-preserving characteristics for achieving topographic, hydrophilic, and antibacterial activation of metal implants without dissolution. Although the reliability and chemical stability of CNW coatings in living organisms still need further experimental verification, the strategy described here provides valuable insights into the surface structural design of advanced multifunction bone implants for future applications in clinic.

## Figures and Tables

**Table 1:** Roughness values based on AFM analysis of P-Ti, A-Ti, SNW/A-Ti, and CNW/A-Ti.

Sample Name	Roughness (nm)		
	Ra	Rq	Rz
P-Ti	141.1 ± 7.4	181.1 ± 4.7	1074.0 ± 97.0
A-Ti	205.9 ± 12.8	256.3 ± 13.8	1180.9 ± 113.4
SNW/A-Ti	239.5 ± 9.9	312.6 ± 11.1	2325.7 ± 94.1
CNW/A-Ti	244.1 ± 12.0	321.0 ± 12.1	2401.0 ± 109.3

## Data Availability

Data are contained within the article.
